# Endothelial NOS (NOS3) impairs myocardial function in developing sepsis

**DOI:** 10.1007/s00395-013-0330-8

**Published:** 2013-02-10

**Authors:** Annette M. van de Sandt, Rainer Windler, Axel Gödecke, Jan Ohlig, Simone Zander, Michael Reinartz, Jürgen Graf, Ernst E. van Faassen, Tienush Rassaf, Jürgen Schrader, Malte Kelm, Marc W. Merx

**Affiliations:** 1 Division of Cardiology, Pneumology and Angiology, Department of Medicine, University Hospital Düsseldorf, Moorenstrasse 5, 40225 Düsseldorf, Germany; 2 Department of Cardiovascular Physiology, Heinrich-Heine-University, Düsseldorf, Germany; 3 Aero Medical Center, Deutsche Lufthansa AG, Frankfurt, Germany; 4 Medical School, Philipps-University Marburg, Marburg, Germany; 5 Department of Nephrology, Leiden University Medical Centre (LUMC), Leiden, The Netherlands; 6 Department of Molecular Cardiology, Heinrich-Heine-University, Düsseldorf, Germany

**Keywords:** Sepsis, Septic cardiomyopathy, Nitric oxide, NOS3

## Abstract

Endothelial nitric oxide synthase (NOS)3-derived nitric oxide (NO) modulates inotropic response and diastolic interval for optimal cardiac performance under non-inflammatory conditions. In sepsis, excessive NO production plays a key role in severe hypotension and myocardial dysfunction. We aimed to determine the role of NOS3 on myocardial performance, NO production, and time course of sepsis development. NOS3^−/−^ and C57BL/6 wildtype mice were rendered septic by cecum ligation and puncture (CLP). Cardiac function was analyzed by serial echocardiography, in vivo pressure and isolated heart measurements. Cardiac output (CO) increased to 160 % of baseline at 10 h after sepsis induction followed by a decline to 63 % of baseline after 18 h in wildtype mice. CO was unaltered in septic NOS3^−/−^ mice. Despite the hyperdynamic state, cardiac function and mean arterial pressure were impaired in septic wildtype as early as 6 h post CLP. At 12 h, cardiac function in septic wildtype was refractory to catecholamines in vivo and respective isolated hearts showed impaired pressure development and limited coronary flow reserve. Hemodynamics remained stable in NOS3^−/−^ mice leading to significant survival benefit. Unselective NOS inhibition in septic NOS3^−/−^ mice diminished this survival benefit. Plasma NO_*x*_- and local myocardial NO_*x*_- and NO levels (via NO spin trapping) demonstrated enhanced NO_*x*_- and bioactive NO levels in septic wildtype as compared to NOS3^−/−^ mice. Significant contribution by inducible NOS (NOS2) during this early phase of sepsis was excluded. Our data suggest that NOS3 relevantly contributes to bioactive NO pool in developing sepsis resulting in impaired cardiac contractility.

**Electronic supplementary material:**

The online version of this article (doi:10.1007/s00395-013-0330-8) contains supplementary material, which is available to authorized users.

## Introduction

In the cardiovascular system, the signaling molecule nitric oxide (NO) has a crucial role in maintaining cardiac performance. Endothelial nitric oxide synthase (eNOS/NOS3) has been identified as the sole NOS isoform participating in mechanosensitive regulation of cardiac function. Under normoxic and non-inflammatory conditions NOS3-derived NO has a positive inotropic and lusitropic effect optimizing cardiac performance and filling (for review see [2, 22]). In the heart, NOS3 is not only expressed in endothelial cells, but also in cardiac myocytes, where it is localized at the peripheral plasmalemma and in t-tubules, in close opposition to the sarcoplasmatic reticulum. Here, it participates in regulation of myocardial metabolism [40] and contractility [49] (for review see also: [2, 22]). Positive inotropic effects of NO at lower concentrations are followed by a negative effect at higher concentrations [33, 53]. NO induced contractile dysfunction is paralleled by a reduction in myocardial energy status [33, 53]. NO is also part of signal transduction of TNF-α in different cardiac diseases [15, 66, 68, 75]. In the setting of myocardial ischemia/reperfusion injury endogenous NO contributes to hibernation [27] and myocardial NOS3 transfection attenuates ischemia/reperfusion injury [63]. Increased cardiac interstitial NO production could be observed during early ischemia and early reperfusion [58], which is in part derived from activated NOS isoforms [19, 25].

In sepsis, excessive production of NO is an important player during hypotension and catecholamine-resistant septic shock [12] and contributes to myocardial dysfunction [45].

Sepsis is the leading cause of death in critically ill patients [30] and severe hypotension and myocardial dysfunction are well-recognized manifestations of organ dysfunction in sepsis [56]. The presence of cardiovascular dysfunction in sepsis is associated with a significantly increased mortality rate of 70–90 % as compared to 20 % in septic patients without cardiovascular impairment [8]. Septic cardiomyopathy is characterized by reversible biventricular dilatation, decreased ejection fraction, and impaired response to fluid resuscitation and catecholamine stimulation [35, 45, 56]. Following the observation that serum from septic patients decreases myocyte contractile function, a number of circulating factors has been studied extensively, indicating myocardial depressant mediators including NO [36, 39, 45].

The inducible NOS (iNOS/NOS2) is generally believed to be the high-capacity NO-producing enzyme in sepsis. NOS2 is expressed in the myocardium [50] and in many other organs on demand and requires several hours to be activated (for review see [23]).

The balance between NOS3 (eNOS) and NOS2 (iNOS) has been the subject of several in vivo studies, showing that endotoxins and cytokines stimulate NOS2 expression, but decrease NOS3 activity (for review see [41]). Cardiomyocyte-specific overexpression of eNOS prevented myocardial dysfunction and death after sepsis induction [31] and eNOS^−/−^ aggravated myocardial dysfunction in the late phase of sepsis [3]. These findings are contradicted by evidence postulating a proinflammatory role for NOS3: it has been demonstrated that NOS3-derived NO plays a crucial role in facilitating NOS2 expression in an in vivo model of endotoxemia, which was reflected by a more stable hemodynamic profile in eNOS^−/−^ mice [7]. eNOS-derived NO exhibited proinflammatory characteristics and contributed to dysfunction of cerebrovascular endothelial cells during early onset of sepsis [21]. On the vascular level, blockade of eNOS activation suppressed LPS-induced iNOS-expression in isolated aortic rings [72].

Given this controversy concerning a proinflammatory role of NOS3 and that NO is intricately involved in regulating cardiovascular function [74], the present study aims to elucidate the influence of NOS3 on myocardial function in sepsis development.

## Methods

### Sepsis induction and unselective NOS inhibition

Male NOS3^−/−^ and C57BL/6 wildtype (WT) mice were rendered septic by cecum ligation and puncture (CLP) [43, 44]. Anesthesia was induced by intraperitoneal (i.p.) administration of ketamine (60 μg/g body weight (BW)) and xylazine (10 μg/g BW). Through a 1-cm abdominal midline incision, the cecum was ligated below the ileo-cecal valve with careful attention to avoid obstruction of the ileum or colon. The cecum was then subjected to a single “through and through” perforation with a 20-gauge needle. After repositioning of the bowel, the abdominal incision was closed in layers with standard silk surgical suture 4-0 (Ethicon, Somerville, NJ, USA). Sham-operated mice underwent the same procedure except for ligation and perforation of the cecum. Animals were handled in compliance with federal regulations, the local ethical committee and the state animal welfare commission. Pain medication (buphrenorphine 0.05 mg/kg BW; Temgesic^®^, Essex Pharma, Grünenthal, Germany) and volume support (NaCl 0.9 %, 0.05 ml/g BW) were administered subcutaneously immediately after sepsis induction and every 8 h thereafter. All mice had unlimited access to food and water. Bacteriologic evaluation of blood and peritoneal lavage fluid obtained at 12 h after CLP confirmed bacterial peritonitis and bacteremia in WT and NOS3^−/−^ mice, whereas no pathogens could be cultured out of blood drawn from sham-operated mice. The pathogens identified were *Escherichia coli*, *Diplococcus*, and *Staphylococcus xylosus*. To achieve unselective NOS inhibition in a subset of the studied animals, osmotic mini pumps secreting *S*-ethylisothiourea (370 μg/h) (ETU) were placed into the peritoneal cavity immediately following sepsis induction.

### Echocardiography

Conscious mice were examined by serial echocardiography [43, 44]. Employing a 15-MHz linear transducer connected to a Sonos 5500™ (Phillips Medical Systems; frequency fusion: 5; interrogation depth 2 cm), the heart was imaged in two-dimensional (2D) mode in the parasternal long axis view and 2D guided M-mode images were obtained at the aortic root for offline aortic diameter measurements. Aortic flow velocity was measured with pulsed-wave Doppler. The reference volume was placed just above the aortic root with careful angle-adjustment strictly parallel to the ascending aorta. Cardiac output was computed from aortic flow velocity time integral, aortic root diameter, and heart rate.

### In vivo hemodynamic measurements

6 and 12 h post-CLP, invasive hemodynamics were assessed using a 1.4F Millar pressure catheter (SPR-839, Millar Instrument, Houston, TX, USA) placed into the left ventricle through the right carotid artery. Pressure data were analyzed with dedicated software (IOX, EMKA, Paris, France) to calculate left-ventricular developed pressure (LVDP), and the first derivatives of left intraventricular pressure (rate of pressure development +d*P*/d*t*_max_ and rate of pressure decrease −d*P*/d*t*_min_). For catecholamine responsiveness, norepinephrine was administered at 0.4 μg/kg/min, i.p. Mean arterial blood pressure was measured in the aorta ascendens.

### Langendorff setup

For isolated heart measurements murine hearts were taken at 6 and 12 h post-CLP, and mounted with retrograde perfusion at 100 mmHg constant pressure with modified Krebs–Henseleit buffer in an isolated heart apparatus (Hugo Sachs Elektronik), as previously described [13, 42–44]. In brief, mice were anesthetized and injected with 250 IU heparin i.p.. The hearts were rapidly excised and transferred for preparation of the aortic arch to oxygenated Krebs–Henseleit buffer. The aorta was cannulated, and hearts were perfused at 100 mmHg constant pressure with modified Krebs–Henseleit buffer containing (in mM) NaCl 116, KCl 4.6, MgSO_4_ 1.1, NaHCO_3_ 24.9, CaCl_2_ 2.5, KH_2_PO_4_ 1.2, glucose 8.3, pyruvate 2.0 and EDTA 0.5, equilibrated with 95 % O_2_ and 5 % CO_2_ (pH 7.4, 37 °C). A home-made fluid-filled polyethylene balloon was inserted into the left ventricle and connected via a fluid-filled polyethylene tubing to a further pressure transducer. Left ventricular end-diastolic pressure was set at 5 mmHg. Hearts were stimulated at 600 beats per minute and were allowed to stabilize for 20 min at 100 mg constant perfusion pressure prior to data acquisition. Left ventricular pressure (LVP), perfusion pressure, aortic flow, and heart rate were measured continuously using a personal computer with analog–digital converter (2,000 Hz) and dedicated software (EMKA Technologies, Paris, France). Derivative parameters (d*P*/d*t*_max_, d*P*/d*t*_min_, coronary flow) were displayed in real time and recorded. Bradykinin and adenosine were infused into the aortic cannula at a concentration of 5 and 1 μmol, respectively.

### Measurement of nitrite/nitrate in plasma and heart tissue

Blood samples were collected by intracardiac puncture at baseline, 6 and 12 h after sepsis induction. Blood and tissue samples were prepared for determination of nitrate and nitrite [52]. Blood samples were centrifuged and plasma was aspirated and stored at −80 °C. To perform analysis, frozen plasma was thawed and mixed with methanol (1:1, v/v) to precipitate the proteins. Samples were centrifuged again and a NO_*x*_-analyzing system (ENO-20 Analyzer, EICOM Corp., Kyoto, Japan) was used to analyze the supernatant. The high-pressure liquid chromatography (HPLC) technique is a highly sensitive technique for measurement of nitrite and nitrate. This method employs ion chromatography with online reduction of nitrate to nitrite and subsequent postcolumn derivatization with the Griess reagent. The detection limit for nitrite and nitrate is 1 nmol/l for either anion at an injection volume of 100 μl [18, 26, 34, 51]. The high validity has been shown as compared to other highly sensitive and specific techniques such as chemiluminescence detection. After thoracotomy, a cannula was inserted into the heart and the heart was flushed free of blood by in situ perfusion with NaCl supplemented with *N*-ethylmaleimide (NEM)/EDTA (100/2.5 mM) at a rate of 10 ml/min. After perfusion, hearts were excised from the mice, snap frozen in liquid nitrogen and homogenized immediately in ice-cold *N*-ethylmaleimide/EDTA-containing perfusion buffer by using a Schuett homogen^plus^ semi automatic glass-on-glass-homogenizer and kept on ice. For determination of nitrite and nitrate (after reaction with nitrate reductase) in biological tissues reductive gas phase chemiluminescence (CLD, Model 77am sp or Model 88am, Eco Physics; Duernten, Switzerland) was used.

### Detection of NO radicals in heart tissue

Local NO levels in murine heart tissue were detected by spin trapping with iron–dithiocarbamate complexes which is one of the most specific methods for NO detection in biological tissue. Na^2+^-DETC was injected intraperitoneally and Fe^2+^-citrate subcutaneously into the scruff of the neck, respectively with final dosages of 500 mg sodium DETC/kg; 35 mg FeSO_4_/kg and 190 mg sodium citrate/kg. The application of iron–dithiocarbamate complexes as spin trap is motivated by their high affinity to bind nitric oxide radicals. Applied spin-trapping reagents react with free radicals to form relatively stable radical adducts that can be detect by EPR spectroscopy. Iron–nitrosyl complexes were generated in cardiac tissue itself: upon their formation the Fe–DETC complexes, being insoluble, remain immobilized in the apolar compartments of the tissues so that the tissue adduct yields of MNIC reflect the local levels of bioavailable NO in that particular tissue type [69, 71]. The NO trapping proceeded for 30 min before sacrifice of the animals. The hearts were excised, incubated in strong HEPES buffer (150 mM, pH 7.4), snap frozen in liquid nitrogen and stored at 77 K. After reduction with dithionite EPR spectra were taken at 77 K on a Bruker ESP300E spectrometer, as described previously [69, 70]. The yields of NO–Fe^2+^–DETC complexes were quantified by comparing with frozen reference solutions of NO–Fe^2+^–MGD in PBS buffer.

### RNA extraction and quantitative RT-PCR analysis

Total RNA was extracted from mouse hearts using “RNeasy^®^ fibrous tissue kit” (Qiagen). Total RNA (1 μg per sample) was reverse-transcribed, and real-time PCR was performed in triplicate using the Applied Biosystems 7300 Fast Real-time PCR system and TaqMan^®^ GenExpression Assays (Applied Biosystems) for NOS3 (nitric oxide synthase 3, endothelial; Mm00435217_m1), NOS2 (nitric oxide synthase 2, inducible; Mm00440502_m1), NOS1 (nitric oxide synthase 1, neuronal; Mm00435175_m1), TNF-α (tumor necrosis factor; Mm00443258_m1), interleukin-6 (Mm00446190_m1) and GAPDH as endogenous control (glyceraldehyde-3-phosphate dehydrogenase; Mm99999915_g1). The setup of reaction consisted of 1 μl of cDNA (10 ng), 1 μl of TaqMan primer set, 10 μl Taq [TaqMan^®^ Gen Expression Master Mix (2×); no. 4369016], and 8 μl of H_2_O. PCR was performed according to manufacturer’s instructions (standard run type).

### Immunohistochemistry and blood cell count

Heart samples were fixed in 4 % formaldehyde phosphate buffer, dehydrated and paraffin-embedded. Serial sections (3 per mouse, 400 μm apart) were stained to analyze the heart tissue for the content of neutrophils (Naphthol AS-D Chloroacetate kit, Sigma), monocytes (α-Naphthyl Acetate kit, Sigma) and lymphocytes (CD3 (Serotec), Universal Strepavidin FITC, Vectors Laboratories and Biotinylated Anti-Mouse/Rabbit/Goat IgG, Vectors Laboratories). Cells were numbered in six different fields per section (cells/mm^2^). For 3-nitrotyrosine detection serial sections were incubated with a polyclonal antibody against nitrotyrosine (USBiological, N2700-06), followed by sheep anti-rabbit IgG conjugated to FITC (Rockland, 611-641-122).

### Statistical analysis

The results are given as mean ± SD. For repeated measurements, data were analyzed by two-way ANOVA followed by Bonferroni’s post hoc test. Otherwise, an unpaired Student’s *t* test was applied. *p* < 0.05 was set as the threshold of significance. Kaplan–Meier survival curves were compared using a log-rank test to determine significance. Myocardial gene expression was analyzed by REST 2009 software (Qiagen).

## Results

### Survival after CLP is profoundly prolonged in NOS3^−/−^ mice

**Fig. 1:**
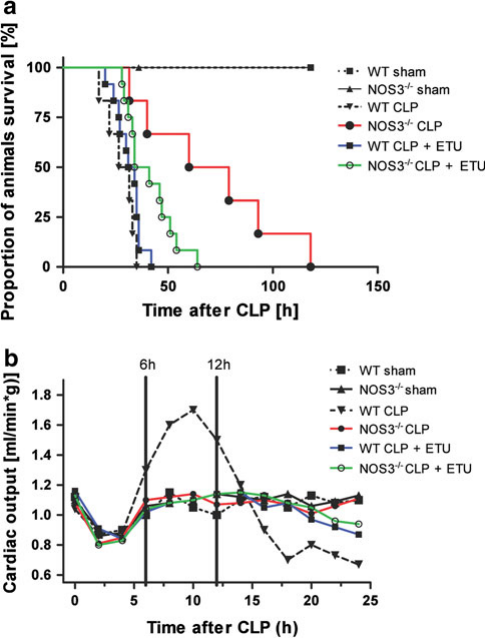
NOS3 induces a hyperdynamic state and impairs survival in sepsis. **a** Prolonged survival in NOS3^−/−^ mice. Survival time of septic NOS3^−/−^ mice was profoundly increased as compared to septic WT mice. Unselective NOS inhibition with ETU diminished survival benefit in septic NOS3^−/−^ mice and led to a slight improvement in septic WT mice. No deaths occurred in sham-operated mice (*n* = 12 per group). **b** Initial hyperdynamic state in septic WT mice. After recovery from anesthesia, cardiac output (CO) increased substantially in WT CLP mice followed by a rapid decline in CO. In contrast, NOS3^−/−^ CLP and all sham-operated animals showed stable CO. CO was also stabilized after unselective NOS inhibition in WT and NOS3^−/−^ mice (*n* = 12 per group)

No deaths occurred in the sham-operated group in both strains. The survival curves for septic mice clearly attest to the prolonged survival in NOS3^−/−^ mice with median survival extended to 70 h from 29 h in WT mice (Fig. 1a). Unselective NOS inhibition with ethylthiourea (ETU) in NOS3^−/−^ mice after sepsis induction diminished this survival benefit, with only a discreet improvement in survival time remaining (mean survival = 38 h, *n* = 12, *p* < 0.05). In contrast to this finding, ETU application in WT mice after CLP led to slight improvement in survival time (mean survival = 31 h, *n* = 12 per group, *p* < 0.05).

### NOS3 induces a hyperdynamic state despite impaired cardiac function

**Fig. 2:**
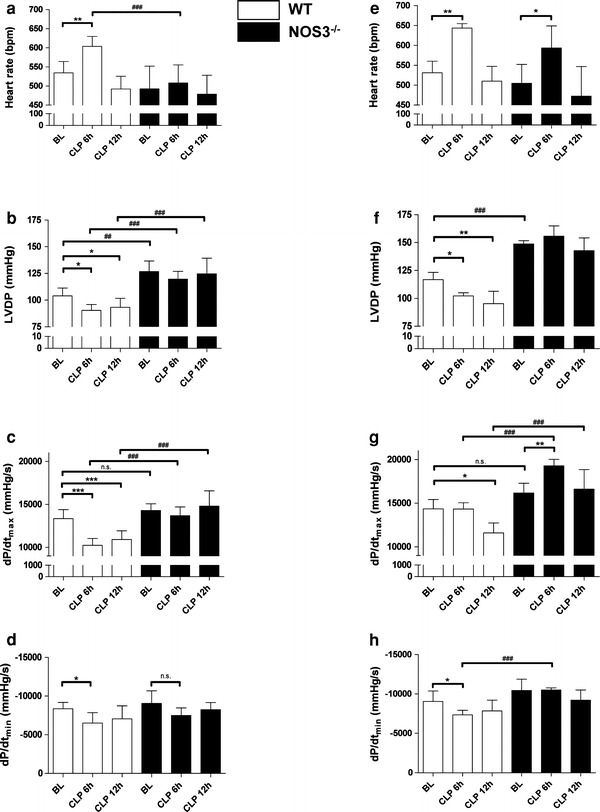
Impaired cardiac function and refractoriness to norepinephrine stimulation in septic WT but not in NOS3^−/−^ mice in developing sepsis (in vivo measurements). Impaired cardiac function evident already at 6 h after sepsis induction with increased heart rate (**a**), decreased LVDP (**b**), d*P*/d*t*_max_ (**c**), and d*P*/d*t*_min_ (**d**) was observed in septic WT, with no difference being detected between septic NOS3^−/−^ mice and non-septic animals. Septic WT mice respond to catecholamine application in early sepsis, which is likely due to increased heart rate (**e**), but remained refractory to norepinephrine stimulation at 12 h post CLP (**f**–**h**) (*p < 0.05, **p < 0.01, ***p < 0.001 vs. WT at baseline; ^##^ *p* < 0.01, ^###^ *p* < 0.001 vs. WT mice; *n* = 4–10 per group; two-way ANOVA followed by Bonferroni’s post hoc test)

**Fig. 3:**
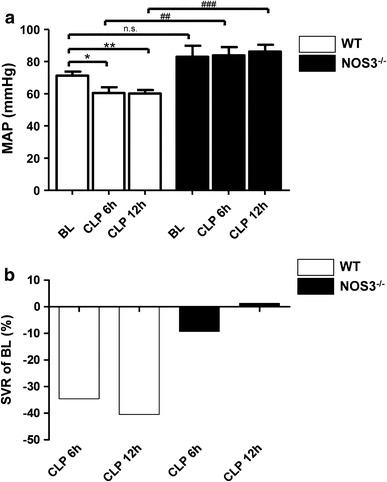
NOS3 induces a drop in systemic vascular resistance. Diminished blood pressure in septic WT, while MAP remained stable in NOS3^−/−^ (*p < 0.05, **p < 0.01 vs. WT at baseline; ^##^ *p* < 0.01, ^###^ *p* < 0.001 vs. WT mice; *n* = 4–10 per group; unpaired student’s *t* test) (**a**). Septic WT mice developed a progressive drop in systemic vascular resistance (**b**) (WT baseline vs. WT CLP 6 h: −34.6 %; WT baseline vs. WT CLP 12 h: −40.5 %). Systemic vascular resistance remained stable in NOS3^−/−^ mice (NOS3^−/−^ baseline vs. NOS3^−/−^ CLP 6 h: −9.2 %, NOS3^−/−^ baseline vs. NOS3^−/−^ CLP 12 h: +1.3 %)

**Fig. 4:**
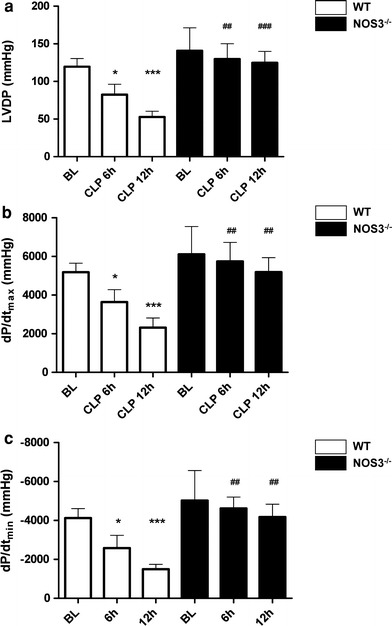
NOS3 exacerbates decreased cardiac output in ongoing sepsis by direct myocardial depression. Ex vivo measurements (**a**–**c**) from isolated hearts of baseline and septic WT and NOS3^−/−^ mice revealed progressive myocardial dysfunction in septic WT mice, which reached the level of significance at 12 h after sepsis induction. NOS3^−/−^ displayed a non-significant tendency towards impaired cardiac function (*p < 0.05, **p < 0.01, ***p < 0.001 vs. WT at baseline; ^#^ *p* < 0.05, ^##^ *p* < 0.01, ^###^ *p* < 0.001 vs. WT mice; *n* = 3–6 per group; two-way ANOVA followed by Bonferroni’s post hoc test)

To address possible changes in cardiac output secondary to sepsis, we studied conscious mice by echocardiography at baseline and every 2 h after sepsis induction (CLP) or sham operation for a total of 24 h. As depicted in Fig. 1b, cardiac output (CO) decreased in all groups during the first 4 h post surgery most likely due to the effects of anesthesia. After recovery from surgery, CO began to increase above baseline values in WT animals subjected to CLP. This increase observed in septic WT mice amounted to a maximum of 160 % of baseline at 10 h after CLP. This hyperdynamic phase was followed by a steady decline in CO, which decreased to values below baseline after 16 h without signs of recuperation. In contrast (subsequent to the first 4 postoperative hours), CO remained stable and unaltered as compared to baseline during the observation period in septic NOS3^−/−^ mice as well as in all sham-operated animals (Fig. 1b). Unselective NOS inhibition led to stable and unaltered cardiac output in septic WT mice without a hyperdynamic phase while having no effect on cardiac output in septic NOS3^−/−^ mice. Corresponding to the beginning of the hyperdynamic phase at 6 h after CLP, cardiac function was additionally assessed invasively using a pressure catheter. At this time point, septic WT mice demonstrated a significant increase in heart rate (WT baseline: heart rate = 547.6 ± 47.3 beats/min; WT CLP 6 h: heart rate = 603.7 ± 26.1 beats/min; WT baseline vs. WT CLP 6 h = *p < 0.05; Fig. 2a), whereas heart rate remained stable in NOS3^−/−^ mice (NOS3^−/−^ baseline: heart rate = 492.5 ± 51.5 beats/min; NOS3^−/−^ CLP 6 h: heart rate = 508.0 ± 47.2 beats/min, NOS3^−/−^ baseline vs. NOS3^−/−^ CLP 6 h = n.s.). Despite the high CO assessed by echocardiography at 6 h post CLP, WT mice exhibited impaired left ventricular function with diminished LVDP and +d*P*/d*t*_max_ as well as impaired −d*P*/d*t*_min_ as compared to animals at baseline (Fig. 2b–d). In contrast, there was no deterioration in LV function detected in septic NOS3^−/−^ mice. With respect to norepinephrine responsiveness, WT mice responded to catecholamine application at 6 h after sepsis induction with increased CO, brought about by an increase in heart rate (Fig. 2e–h). At 12 h post CLP septic WT remained refractory to norepinephrine stimulation with unaltered LVDP, +d*P*/d*t*_max_ and −d*P*/d*t*_min_ while these parameters increased in NOS3^−/−^ mice to an extent comparable to animals at baseline (Fig. 2e–h). When compared with baseline and NOS3^−/−^ mice, mean arterial blood pressure was diminished in septic WT at 6 h after sepsis induction (Fig. 3a). Systemic vascular resistance was estimated from the median mean arterial blood pressure and median cardiac output (Fig. 3b). Although septic WT mice developed a progressive drop in systemic vascular resistance (WT baseline vs. WT CLP 6 h: −34.6 %; WT baseline vs. WT CLP 12 h: −40.5 %) systemic vascular resistance remained stable in NOS3^−/−^ mice (NOS3^−/−^ baseline vs. NOS3^−/−^ CLP 6 h: −9.2 %, NOS3^−/−^ baseline vs. NOS3^−/−^ CLP 12 h: +1.3 %). Although the profoundly reduced vascular resistance in WT took center stage in the initial phase of sepsis (6 h), myocardial dysfunction was evident at 12 h after sepsis induction with impaired LVDP, +d*P*/d*t*_max_ and −d*P*/d*t*_min_ in isolated hearts of septic WT mice. Again, we observed no significant deterioration in cardiac function in isolated hearts of septic NOS3^−/−^ compared to baseline (Fig. 4a–c).

### NOS3 impairs coronary flow reserve

**Fig. 5:**
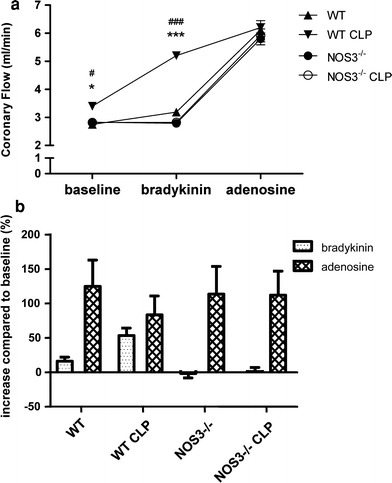
NOS3 impairs coronary flow reserve. Baseline coronary flow (CF) was increased solely in WT CLP mice. After NOS3 stimulation by bradykinin, coronary flow increased most substantially in WT CLP mice with no effect observed in NOS3^−/−^ mice. Adenosine stimulation led to a maximum CF increase in all mice except WT CLP mice, in which CF response to adenosine was not greater than to bradykinin due to impaired coronary flow reserve (*p < 0.05 WT vs. WT CLP at baseline; ***p < 0.001 WT vs. WT CLP after application of bradykinin; ^#^ *p* < 0.05 WT CLP vs. NOS3^−/−^ CLP at baseline, ^###^ *p* < 0.001 WT CLP vs. NOS3^−/−^ CLP after bradykinin application; *n* = 10 per group; two-way ANOVA followed by Bonferroni’s post hoc test)

Coronary flow was increased by 30 % in WT CLP mice as compared to WT mice. No significant difference was detected in coronary flow between septic NOS3^−/−^ and non-septic NOS3^−/−^ animals. Susceptibility to NOS3 stimulation by bradykinin was approximately twice as pronounced in WT CLP mice as compared to non-septic mice, indicating a high level of NOS3 activation secondary to sepsis (Fig. 5a, b). As expected, bradykinin stimulation had no effect in NOS3^−/−^ hearts. Coronary flow reserve measured after adenosine application exhibited the opposite pattern, being diminished in septic WT mice as compared to baseline, while hearts from septic NOS3^−/−^ mice showed close to twofold augmentation of coronary flow similar to the baseline observations (Fig. 5a, b).

### NOS3 contributes to bioactive NO pool

**Fig. 6:**
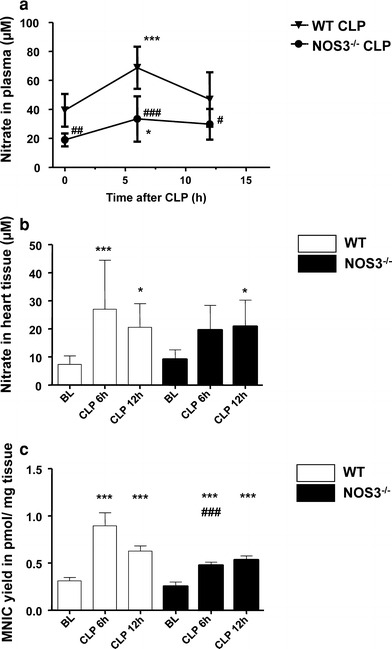
NOS3 contributes to bioactive NO pool in developing sepsis. Plasma nitrate levels (**a**) were increased 6 h after sepsis induction in WT and NOS3^−/−^ mice (***p < 0.001 vs. baseline mice of the same genotype; ^###^ *p* < 0.001 vs. WT mice; *n* = 10–16 per group; two-way ANOVA followed by Bonferroni’s post hoc test). Nitrate levels were lower in NOS3^−/−^ compared to WT mice at baseline. Heart tissue nitrate levels (*n* = 4 per group) (**b**) and bioactive NO levels (**c**) (measured as MNIC yields per pmol wet heart tissue) were increased 6 h after sepsis induction in WT and NOS3^−/−^ mice (**p < 0.05 vs. baseline mice of the same genotype; ***p < 0.001 vs. baseline mice of the same genotype; ^###^ *p* < 0.001 vs. WT mice; *n* = 6–9 per group; two-way ANOVA followed by Bonferroni’s post hoc test), with a prominent increase in septic WT compared to NOS3^−/−^. NO_*x*_- and bioactive NO levels were comparable between both strains at baseline

NO has a short half life of a few ms in vivo, making direct measurement a challenging task in plasma and tissues. Plasma concentrations of downstream metabolites like nitrate and nitrite have been used as proxies for NO proper [47]. Plasma nitrate and nitrite levels were measured at baseline, 6 and 12 h post-CLP using a ${\text{NO}}_{x^-}$ analyzing system. When compared with plasma nitrate levels at baseline, septic WT mice exhibited increased nitrate levels at 6 h after sepsis induction (Fig. 6a). Septic NOS3^−/−^ mice showed a lesser increase in nitrate levels, with baseline values being significantly reduced as compared to WT mice, likely due to the absence of NOS3. Similarly to nitrate plasma levels (Fig. 6a), nitrate increased significantly at 6 h after sepsis induction in the heart tissue of septic WT mice. In contrast, in septic NOS3^−/−^ hearts, we observed a lesser increase in nitrate levels with no significant changes compared to baseline at 6 h post CLP (Fig. 6b). During further progress of sepsis nitrate levels in plasma and heart tissue decreased in septic WT mice. Nitrite levels in plasma and heart tissue were found to be comparable over time and in both strains (data not shown). We used NO spin trapping and EPR to quantify the in vivo NO levels in heart tissue. After in vivo spin trapping with Fe–DETC complexes, EPR spectroscopy of frozen heart tissue showed the characteristic triplet spectrum of ferrous mononitrosyl iron complexes [MNIC, NO–Fe^2+^–(DETC)_2_] in the hearts of NOS3^−/−^ and WT mice. At 6 h post-CLP, the cardiac tissues of septic WT and NOS3^−/−^ mice displayed higher MNIC yields compared to animals at baseline, while the heart tissue of septic NOS3^−/−^ mutants contained significantly lower MNIC concentrations than those of septic WT mice. WT and NOS3^−/−^ mice had significantly lower and comparable yields at baseline (Fig. 6c). Immunohistochemical staining demonstrated an increase of nitrotyrosine in heart tissue of septic WT mice at 6 h post CLP, whereas no differences could be detected in NOS3^−/−^ mice (see Online Resource Figure 1 in the online supplement). Quantitative RT-PCR analysis of NOS2- and NOS3-expression in the hearts at baseline, 6 and 12 h after sepsis induction revealed neither increase in NOS2- nor NOS3-expression in septic WT and NOS3^−/−^ mice compared to baseline (see Online Resource Figure 2a, b). There was no difference detected between both strains. In addition, we analyzed protein expression of NOS3, NOS2, and neuronal NOS (NOS1) in heart tissues. When compared with baseline, NOS3 expression did not differ in septic WT mice at 6 and 12 h post CLP. As it would be expected, western blot revealed the absence of NOS3 (eNOS) protein in NOS3^−/−^ mice [16] (see Online Resource Figure 2c, d). Cardiac protein expression of NOS2 and NOS1 always remained below detection levels in both strains. Determination of phospho-NOS3 revealed no increase in WT mice during sepsis development (see Online Resource Figure 2e). Furthermore, global protein glutathionylation [55] was not increased in hearts of septic mice and signal intensity remained constant at the level of NOS3 suggesting no increase in NOS3 glutathionylation in septic WT mice (see Online Resource Figure 3). There were no signs of increased ROS production in the latter (see Online Resource Figure 4).

### Myocardial inflammatory response

CLP promoted invasion of the myocardium by inflammatory cells (neutrophils, monocytes and lymphocytes) in septic WT and NOS3^−/−^ mice. When compared with septic WT mice the myocardial infiltration with neutrophils and monocytes in NOS3^−/−^ mice was less severe than in WT mice at 12 h post CLP (Table 1). Blood cell count demonstrated a progressive reduction in white blood cell counts in both strains. We further examined the expression levels of genes encoding inflammatory cytokines in the heart at 6 and 12 h after CLP. Levels of TNF-α and IL-6 were increased already at 6 h after sepsis induction in both genotypes.

**Table 1:** Inflammatory response in developing sepsis

Parameter	WT			NOS3^−/−^		
	BL	CLP 6 h	CLP 12 h	BL	CLP 6 h	CLP 12 h
Blood cell count (10^3^/µl)						
White blood cells	5.17 ± 1.64	2.11 ± 0.60***	1.31 ± 0.42***	3.74 ± 0.67^##^	2.60 ± 0.75**	1.86 ± 0 51***
Granulocytes	1.17 ± 0.29	1.53 ± 0.51	0.88 ± 0.27	1.18 ± 0.17	1.78 ± 0.58**	1.04 ± 0.26
Monocytes	0.38 ± 0.17	0.13 ± 0.12***	0.12 ± 0.04***	0.29 ± 0.06	0.14 ± 0.05**	0.16 ± 0.07**
Lymphocytes	3.62 ± 1.20	0.73 ± 0.32***	0.52 ± 0.13***	2.28 ± 0.49^###^	0.68 ± 0.19***	0.63 ± 0.29***
Myocardial histology (cells/mm^2^)						
Granulocytes	755 ± 134	1,778 ± 90***	2,362 ± 78***	566 ± 90	1,559 ± 238***	1,620 ± 206***
Monocytes	896 ± 87	2,297 ± 324***	2,238 ± 229***	645 ± 149	1,538 ± 265***^,###^	1,668 ± 297***
Lymphocytes	439 ± 64	1,375 ± 247***	1,574 ± 411***	318 ± 82	1,272 ± 379***	1,325 ± 233***
Myocardial gene expression						
IL-6	1.0	81.0***	16.7***	1.1	79.1***	6.7
TNF-α	1.0	6.5***	1.4	1.4	6.7***	4.0

Mean ± SD; blood cell count *n* = 6–13 per group; myocardial histology *n* = 4–8 per group; myocardial gene expression *n* = 3 per group

Two-way ANOVA followed by Bonferroni posthoc test; myocardial gene expression was analyzed by REST 2009 software (Quiagen)

* *p* < 0.05 vs. baseline of the same genotype

** *p* < 0.01 vs. baseline of the same genotype

*** *p* < 0.001 vs. baseline of the same genotype

^##^ *p* < 0.01 vs. WT mice

^###^ *p* < 0.001 vs. WT mice

## Discussion

The main finding of the present study is that endothelial NOS (eNOS/NOS3) plays a key role in the development of sepsis. NOS3 facilitates a drop in mean arterial blood pressure and systemic vascular resistance. Furthermore, we observed a hyperdynamic state despite impaired left ventricular function followed by rapid deterioration of cardiac output (CO) and limited coronary flow reserve, leading to short survival times. In contrast, septic NOS3^−/−^ mice display stable blood pressure, preserved coronary flow reserve and CO with survival time extended more than twofold. As possible contributing mechanisms we demonstrate decreased endogenous NO_*x*_-levels and dampened myocardial inflammatory response in an early stage of polymicrobial sepsis.

### NOS3 contributes to bioactive NO pool

That NOS3 contributes to endogenous NO production in developing sepsis is supported by our finding that NOS3^−/−^ mice exhibited a significantly smaller increase of plasma nitrate levels compared to WT during sepsis development. In concordance with our findings, LPS-treatment of WT mice resulted in a profound elevation of plasma NO_*x*_ measurements [7, 72]. This response to LPS in WT was twice as pronounced compared to the increase observed in eNOS^−/−^ animals [7, 72]. In patients who develop septic shock, plasma NO_*x*_ levels and NO_*x*_ concentrations are higher in non-survivors [28]. As a consequence of significantly enhanced endogenous NO production we observed a reduced sensitivity to the NO donor SNP in isolated aortic rings from septic WT mice (see Online Resource Figure 5a). In concordance with our results, relaxation response to NO donors in aortic rings was suppressed in LPS-treated WT mice and unaffected in rings of eNOS^−/−^ animals [72]. In this context, as readout for bioactive NO levels and focussing on the local NO status of cardiac tissue, we detected significantly increased myocardial MNIC yields in septic WT mice compared to septic NOS3^−/−^ animals.

Despite increased endogenous NO production, we did not observe an increase in NOS1-, NOS2-, NOS3-mRNA, as well as NOS1-, NOS2-, NOS3- and phospho-NOS3-protein expression in developing sepsis (Online Resource Figure 2). However, direct evidence for a high level of NOS3 activation is provided by our studies of the septic coronary vasculature, in which baseline flow and response to NOS3 stimulation were markedly increased and coronary flow reserve reduced by a third. No alterations in coronary flow were noted in NOS3^−/−^ mice. An increased eNOS activity has been demonstrated in the initial phase of sepsis [37], followed by induction of iNOS in the later phase [67]. A further study revealed a maximum of iNOS-protein content at 24 h after CLP [11]. In an ovine sepsis model, total NOS increased at 12 and 24 h after injury, whereas iNOS activity was not altered significantly [37]. This was accompanied by an early increase of 3-nitrotyrosine, a marker of protein nitration [37] suggesting that constitutive NOS (NOS3 and NOS1) is the main contributor to increased NO levels in this model. In concordance with this finding, we also detected augmented 3-nitrotyrosine levels in septic WT mice at 6 h after sepsis induction (Online Resource 1). It is known that increased nitrotyrosine formation during sepsis may uncouple NOS3 activity and increase oxidative stress [65]. Superoxide produced by NADPH oxidase, which is considered to be a major source of ROS that are implicated in the pathophysiology of many cardiovascular diseases [59, 76], may react with NO, thereby generating peroxynitrite. The latter is considered to induce delayed and irreversible cardiac damage [57]. In pressure-overloaded ischemic-reperfused hearts, increased nitrosative and oxidative stress contributes to the exacerbating impact of pressure-overload on MPT pore opening and cell death [46]. NOS3 itself can be a superoxide source, thereby causing endothelial dysfunction [14]. Endothelial dysfunction, a key contributor to organ failure and death in sepsis [1, 9], is also clearly visible in septic wildtype mice (Online Resource Figure 5b). In our present study, cardiac tissue of septic wildtype mice displayed no evidence for increased global protein glutathionylation (Online Resource Figure 3) or ROS generation (Online Resource Figure 4) in early sepsis suggesting that the observed hemodynamic alterations are primarily due to increased bioactive NO and nitrosative stress in this early stage of sepsis development.

### NOS3 impairs cardiovascular function in developing sepsis

The observed hemodynamic alterations in our study are typically found also in septic patients, namely a hyperdynamic followed by a hypodynamic state (Fig. 1b). A prominent feature in this context is the reduced vascular tone and vasodilatation in developing sepsis. In concordance, we demonstrate a drop in mean arterial blood pressure and systemic vascular resistance in septic wildtype (WT) mice at 6 h after sepsis induction. The inducible NOS (iNOS/NOS2) is generally believed to be responsible for the hypotension and loss of vascular tone in ongoing sepsis. After LPS treatment, a drop in blood pressure is commonly observed in WT mice, but absent in iNOS^−/−^ mice [20]. Selective inhibitors of iNOS increased mean arterial blood pressure [32] and abrogated LPS-induced loss of vascular tone [60]. Thus, NOS2 appears to be crucial in hypotension in LPS-induced sepsis. In this context, the stable mean arterial blood pressure and systemic vascular resistance in septic NOS3^−/−^ as compared to WT mice observed in the present study might appear unexpected. NOS3-derived NO plays an important role in the regulation of regional vascular tone and blood flow and the constant, low rate of NO production by constitutive NOS forms can be increased acutely as part of the acute inflammatory response [23]. In addition, the activity of NOS3 is required for the induction of NOS2: a temporal reduction in iNOS expression and activity could be demonstrated in LPS-treated septic eNOS^−/−^ mice [7]. Similar to our results, a stable blood pressure profile of eNOS^−/−^ mice has been described during endotoxemia [7]. Following LPS infusion, a markedly increased myocardial eNOS activity has been demonstrated leading to a decrease in blood pressure after 30 min [10]. Concurrent with stable blood pressure and systemic vascular resistance, NOS3^−/−^ mice exhibited preserved LVDP, +d*P*/d*t*_max_ and −d*P*/d*t*_min_ while cardiac function was impaired in septic WT mice already at 6 h after sepsis induction. This time point corresponded to the beginning of the hyperdynamic phase in WT mice. This suggests that the cardiac output increase in septic WT mice is predominantly related to the profoundly reduced systemic vascular resistance due to sepsis-induced vasodilatation and obscures myocardial dysfunction [45], which subsequently becomes evident.

Cardiac function of isolated hearts from septic WT mice was compromised with impaired LVDP, +d*P*/d*t*_max_ and −d*P*/d*t*_min_ at 12 h after sepsis induction. This time point in sepsis development was also characterized by refractoriness to norepinephrine stimulation in vivo. Thus, in this phase of ongoing sepsis, decreased cardiac output may reflect direct myocardial depression. Deviating from this pattern well known from several forms of human sepsis [45, 48], septic NOS3^−/−^ mice exhibited stable CO and preserved cardiac function. NOS3 is considered to be the major isoform in the healthy heart and is expressed in vascular endothelium and cardiac myocytes [2]. It has been suggested that local NO levels in cardiac tissue are important modulators of cardiac contractility, and that high NO levels induce cardiac depression [33, 53]. Increased NOS3-expression in ventricular myocardium of failing human hearts may contribute to contractile dysfunction [61]. Hearts of mice with myocyte-specific eNOS-overexpression had reduced basal contractility that was partially reversed by NOS blockade [4].

Septic NOS3^−/−^ mice exhibited preserved cardiac function, including responsiveness to catecholamine stimulation. When considering the well-described anti-adrenergic effect of NO in the heart [17, 54], the preserved responsiveness to catecholamine stimulation in NOS3^−/−^ mice is probably also due to diminished endogenous NO production. In contrast to the findings from the present study, mice with cardiomyocyte-specific overexpression of eNOS were protected from myocardial dysfunction and death during endotoxemia [31]. In a recent study, septic eNOS^−/−^ mice exhibited more marked myocardial dysfunction 22 h after sepsis induction, which equated to a late phase of sepsis in the model studied [3]. Impaired cardiac function at this time point was associated with an exaggerated inflammatory response [3]. Reasons for these discrepant findings are probably multi-factorial and depend amongst others on the sepsis model used: Although our model demonstrated a peak in TNF-α and IL-6 expression at 6 h post CLP in both strains, Bougaki et al. [3] observed an increase in TNF-α as well as IL-6 expression at 22 h after colon ascendens stent peritonitis and genetic eNOS deficiency was associated with enhanced induction of proinflammatory cytokines. In contrast to this study, a proinflammatory role of NOS3 was postulated by Connelly et al. [7]. Fitting well with initial proinflammatory effects of NOS3, we observed that WT mice exhibited a more marked myocardial infiltration with neutrophils, monocytes, and lymphocytes in the hearts at 12 h after sepsis induction. The activation and recruitment of inflammatory cells to the heart is a major early event in cardiac dysfunction promoted by septicemia [64]. Thus, dampened myocardial inflammatory response and decreased endogenous NO_*x*_ levels may contribute to cardiovascular stabilization in the present model.

Interestingly, unselective NOS inhibition also led to stabilization of CO in septic WT mice. Despite stabilized CO, septic WT mice exhibited just a minor improvement in survival time after unselective NOS inhibition. This is in concordance with the conflicting and overall disappointing results obtained by unselective NOS inhibition in septic patients [5, 38]. With a large amount of data supporting a detrimental role for NOS2 in sepsis [24, 29, 62], it might be expected that NOS2 inhibition would result in additional survival benefit in NOS3^−/−^ mice. In contrast to this expectation, our studies involving unselective NOS inhibition in NOS3^−/−^ mice demonstrate significantly reduced survival time. iNOS2^−/−^ mice have been shown to have decreased survival in a CLP model of sepsis [6] and decreased defense against bacterial inoculation in iNOS^−/−^ mice has been demonstrated [73]. In conclusion, our results support that NOS3 is a major source of endogenous NO in developing sepsis leading to myocardial dysfunction.

## Electronic supplementary material

Below is the link to the electronic supplementary material.

Supplementary material 1 (PDF 13178 kb)

Supplementary material 2 (PDF 1273 kb)

Supplementary material 3 (PDF 795 kb)

Supplementary material 4 (PDF 71 kb)

Supplementary material 5 (PDF 113 kb)

## References

[1] Aird WC (2003). The role of the endothelium in severe sepsis and multiple organ dysfunction syndrome. Blood.

[2] Balligand JL, Feron O, Dessy C (2009). eNOS activation by physical forces: from short-term regulation of contraction to chronic remodeling of cardiovascular tissues. Physiol Rev.

[3] Bougaki M, Searles RJ, Kida K, Yu J, Buys ES, Ichinose F (2010). Nos3 protects against systemic inflammation and myocardial dysfunction in murine polymicrobial sepsis. Shock.

[4] Brunner F, Andrew P, Wölkert G, Zechner R, Mayer B (2001). Myocardial contractile function and heart rate in mice with myocyte-specific overexpression of endothelial nitric oxide synthase. Circulation.

[5] Cobb JP (2001). Nitric oxide synthase inhibition as therapy for sepsis: a decade of promise. Surg Infect.

[6] Cobb JP, Hotchkiss R, Swanson PE et al (1999) Inducible nitric oxide synthase (iNOS) gene deficiency increases the mortality of sepsis in mice. Surgery 126:438–442. pii: N9c41cb98N2a1153cc 10455918

[7] Connelly L, Madhani M, Hobbs AJ (2005). Resistance to endotoxic shock in endothelial nitric-oxide synthase (eNOS) knock-out mice: a pro-inflammatory role for eNOS-derived no in vivo. J Biol Chem.

[8] Court O, Kumar A, Parrillo JE, Kumar A (2002). Clinical review: myocardial depression in sepsis and septic shock. Crit Care.

[9] Davis JS, Darcy CJ, Yeo TW, Jones C, McNeil YR, Stephens DP, Celermajer DS, Anstey NM (2011). Asymmetric dimethylarginine, endothelial nitric oxide bioavailability and mortality in sepsis. PLoS ONE.

[10] Doursout MF, Oguchi T, Fischer UM, Liang Y, Chelly B, Hartley CJ, Chelly JE (2008). Distribution of NOS isoforms in a porcine endotoxin shock model. Shock.

[11] Fernandes D, Sordi R, Kramer-Pacheco LK, Nardi GM, Heckert BT, Villela CG, Lobo AR, Barja-Fidalgo C, Assreuy J (2009). Late, but not early, inhibition of soluble guanylate cyclase decreases mortality in a rat sepsis model. J Pharmacol Exp Ther.

[12] Flierl MA, Rittirsch D, Huber-Lang MS, Sarma JV, Ward PA (2008). Molecular events in the cardiomyopathy of sepsis. Mol Med.

[13] Flögel U, Decking UK, Gödecke A, Schrader J (1999). Contribution of NO to ischemia-reperfusion injury in the saline-perfused heart: a study in endothelial NO synthase knockout mice. J Mol Cell Cardiol.

[14] Förstermann U, Münzel T (2006). Endothelial nitric oxide synthase in vascular disease: from marvel to menace. Circulation.

[15] Garlie JB, Hamid T, Gu Y, Ismahil MA, Chandrasekar B, Prabhu SD (2011). Tumor necrosis factor receptor 2 signaling limits ß-adrenergic receptor-mediated cardiac hypertrophy in vivo. Basic Res Cardiol.

[16] Gödecke A, Decking UK, Ding Z, Hirchenhain J, Bidmon H-J, Gödecke S, Schrader J (1998). Coronary hemodynamics in endothelial NO synthase knockout mice. Circ Res.

[17] Godecke A, Heinicke T, Kamkin A, Kiseleva I, Strasser RH, Decking UK, Stumpe T, Isenberg G, Schrader J (2001). Inotropic response to beta-adrenergic receptor stimulation and anti-adrenergic effect of ACh in endothelial NO synthase-deficient mouse hearts. J Physiol.

[18] Grau M, Hendgen-Cotta UB, Brouzos P, Drexhage C, Rassaf T, Lauer T, Dejam A, Kelm M, Kleinbongard P (2007). Recent methodological advances in the analysis of nitrite in the human circulation: nitrite as a biochemical parameter of the l-arginine/NO pathway. J Chromatogr B Analyt Technol Biomed Life Sci.

[19] Guo Y, Sanganalmath SK, Wu W, Zhu X, Huang Y, Tan W, Ildstad ST, Li Q, Bolli R (2012). Identification of inducible nitric oxide synthase in peripheral blood cells as a mediator of myocardial ischemia/reperfusion injury. Basic Res Cardiol.

[20] Hallemeesch MM, Janssen BJA, de Jonge WJ, Soeters PB, Lamers WH, Deutz NEP (2003). NO production by cNOS and iNOS reflects blood pressure changes in LPS-challenged mice. Am J Physiol Endrocrinol Metab.

[21] Handa O, Stephen J, Cepinskas G (2008). Role of endothelial nitric oxide synthase-derived nitric oxide in activation and dysfunction of cerebrovascular endothelial cells during early onsets of sepsis. Am J Physiol Heart Circ Physiol.

[22] Hare JM, Stamler J (2005). NO/redox disequilibrium in the failing heart and cardiovascular system. J Clin Invest.

[23] Hauser B, Bracht H, Matejovic M, Radermacher P, Venkatesh B (2005). Nitric oxide synthase inhibition in sepsis? Lessons learned from large-animal studies. Anesth Analg.

[24] Heemskerk S, Masereeuw R, Russel FG, Pickkers P (2009). Selective iNOS inhibition for the treatment of sepsis-induced acute kidney injury. Nat Rev Nephrol.

[25] Heinzel FR, Gres P, Boengler K, Duschin A, Konietzka I, Rassaf T, Snedovskaya J, Meyer S, Skyschally A, Kelm M, Heusch G, Schulz R (2008). Inducible nitric oxide synthase expression and cardiomyocyte dysfunction during sustained moderate ischemia in pigs. Circ Res.

[26] Hendgen-Cotta U, Grau M, Rassaf T, Kelm M, Kleinbongard P (2008). Reductive gas-phase chemiluminescence and flow injection analysis for measurement of the nitric oxide pool in biological matrices. Methods Enzymol.

[27] Heusch G, Post H, Michel MC, Kelm M, Schulz R (2000). Endogenous nitric oxide and myocardial adaptation to ischemia. Circ Res.

[28] Ho JT, Chapman MJ, O′Conner S, Lam S, Edwards J, Ludbrook G, Lewis JG, Torpy DJ (2010) Characteristics of plasma NO_*x*_ levels in severe sepsis: high interindividual variability and correlation with illness severity, but lack of correlation with cortisol levels. Clin Endocrinol (Oxf) 73:413–420. doi:10.1111/j.1365-2265.2010.03817.x 10.1111/j.1365-2265.2010.03817.x 20455885

[29] Hollenberg SM, Broussard M, Osman J, Parrillo JE (2000). Increased microvascular reactivity and improved mortality in septic mice lacking inducible nitric oxide synthase. Circ Res.

[30] Hotchkiss RS, Karl IE (2003). The pathophysiology and treatment of sepsis. N Engl J Med.

[31] Ichinose F, Buys ES, Neilan TG, Furutani EM, Morgan JG, Jassal DS, Graveline AR, Searles RJ, Lim CC, Kaneki M, Picard MH, Scherrer-Crosbie M, Janssens S, Liao R, Bloch KD (2007). Cardiomyocyte-specific overexpression of nitric oxide synthase 3 prevents myocardial dysfunction in murine models of septic shock. Circ Res.

[32] Kadoi Y, Goto F (2004) Selective inducible nitric oxide inhibition can restore hemodynamics, but does not improve neurological dysfunction in experimentally-induced septic shock in rats. Anesth Analg 99:212–220. doi:10.1213/01ANE.0000118111.94913.22 10.1213/01.ANE.0000118111.94913.22 15281532

[33] Kelm M, Schäfer S, Dahmann R, Dolu B, Perings S, Decking U, Schrader J, Strauer BE (1997). Nitric oxide induced contractile dysfunction is related to a reduction in myocardial energy generation. Cardiovasc Res.

[34] Kleinbongard P, Dejam A, Lauer T, Rassaf T, Schindler A, Picker O, Scheeren T, Gödecke A, Schrader J, Schulz R, Heusch G, Schaub GA, Bryan NS, Feelisch M, Kelm M (2003). Plasma nitrite reflects constitutive nitric oxide synthase activity in mammals. Free Radic Biol Med.

[35] Kumar A, Haery C, Parrillo JE (2001). Myocardial dysfunction in septic shock: part I. Clinical manifestation of cardiovascular dysfunction. J Cardiothorac Vasc Anesth.

[36] Kumar A, Krieger A, Symeoneides S, Kumar A, Parrillo JE (2001). Myocardial dysfunction in septic shock: part II. Role of cytokines and nitric oxide. J Cardiothorac Vasc Anesth.

[37] Lange M, Connelly R, Traber DL, Hamahata A, Nakano Y, Esechie A, Jonkam C, von Borzyskowski S, Traber LD, Schmalstieg FC, Herndon DN, Enkhbaatar P (2010). Time course of nitric oxide synthases, nitrosative stress, and poly(ADP ribosylation) in an ovine sepsis model. Crit Care.

[38] Lopez A, Lorente JA, Steingrub J, Bakker J, McLuckie A, Willatts S, Brockway M, Anzueto A, Holzapfel L, Breen D, Silverman JT, Donaldson J, Arneson C, Grove G, Grossman S, Grover R (2004). Multiple-center, randomized, placebo-controlled, double-blind study of the nitric oxide synthase inhibitor 546C88: effect on survival in patients with septic shock. Crit Care Med.

[39] Lupia E, Spatola T, Cuccurullo A, Bosco O, Mariano F, Pucci A, Ramella R, Alloatti G, Montrucchio G (2010). Thrombopoietin modulates cardiac contractility in vitro and contributes to myocardial depressing activity of septic shock serum. Basic Res Cardiol.

[40] Martin C, Schulz R, Post H, Gres P, Heusch G (2003). Effect of NO synthase inhibition on myocardial metabolism during moderate ischemia. Am J Physiol Heart Circ Physiol.

[41] McGown CC, Brookes ZL (2007). Beneficial effects of statins on the microcirculation during sepsis: the role of nitric oxide. Br J Anaesth.

[42] Merx MW, Flogel U, Stumpe T, Gödecke A, Decking UK, Schrader J (2001). Myoglobin facilitates oxygen diffusion. FASEB J.

[43] Merx MW, Liehn EA, Graf J, van de Sandt A, Schaltenbrand M, Schrader J, Hanrath P, Weber C (2005). Statin treatment after onset of sepsis in a murine model improves survival. Circulation.

[44] Merx MW, Liehn EA, Janssens U, Lütticken R, Schrader J, Hanrath P, Weber C (2004). HMG-CoA reductase inhibitor simvastatin profoundly improves survival in a murine model of sepsis. Circulation.

[45] Merx MW, Weber C (2007). Sepsis and the heart. Circulation.

[46] Mozaffari MS, Baban B, Liu JY, Abebe W, Sullivan JC, El-Marakby A (2011) Mitochondrial complex I and NAD(P)H oxidase are major sources of exacerbated oxidative stress in pressure-overloaded ischemic-reperfused hearts. Basic Res Cardiol 106:287–297. doi:10.1007/s00395-011-0150-7 10.1007/s00395-011-0150-7 21246205

[47] Nussler AK, Bruckner UB, Vogt J, Radermacher P (2002). Measuring end products of nitric oxide in vivo. Methods Enyzmol.

[48] Parrillo JE (1993). Pathogenetic mechanisms of septic shock. N Engl J Med.

[49] Post H, Schulz R, Gres P, Heusch G (2001). No involvement of nitric oxide in the limitation of beta -adrenergic inotropic responsiveness during ischemia. Am J Physiol Heart Circ Physiol.

[50] Preiser JC, Zhang H, Vray B, Hrabak A, Vincent JL (2001). Time course of inducible nitric oxide synthase activity following endotoxin administration in dogs. Nitric Oxide.

[51] Rassaf T, Bryan NS, Kelm M, Feelisch M (2002). Concomitant presence of *N*-nitroso and *S*-nitroso proteins in human plasma. Free Radic Biol Med.

[52] Rassaf T, Feelisch M, Kelm M (2004). Circulating NO pool: assessment of nitrite and nitroso species in blood and tissues. Free Radic Biol Med.

[53] Rassaf T, Poll LW, Brouzos P, Lauer T, Totzeck M, Kleinbongard P, Gharini P, Andersen K, Schulz R, Heusch G, Mödder U, Kelm M (2006). Positive effects of nitric oxide on left ventricular function in humans. Eur Heart J.

[54] Reinartz M, Molojavyi A, Moellendorf S, Hohlfeld T, Heger J, Gödecke A (2011). ß-Adrenergic signaling and response to pressure overload in transgenic mice with cardiac-specific overexpression of inducible NO synthase. Nitric Oxide.

[55] Reinartz M, Ding Z, Flögel U, Gödecke A, Schrader J (2008). Nitrosative stress leads to protein glutathiolation, increased S-nitrosation and up-regulation of peroxiredoxins in the heart. J Biol Chem.

[56] Rudiger A, Singer M (2007) Mechanisms of sepsis-induced cardiac dysfunction. Crit Care Med 35:1599–1608. doi:10.1097/01.CCM.0000266683.64081.02 10.1097/01.CCM.0000266683.64081.02 17452940

[57] Schulz R, Dodge KL, Lopaschuk GD, Clanachan AS (1997). Peroxynitrite impairs cardiac contractile function by decreasing cardiac efficiency. Am J Physiol.

[58] Schulz R, Kelm M, Heusch G (2004). Nitric oxide in myocardial ischemia/reperfusion injury. Cardiovasc Res.

[59] Sirker A, Zhang M, Shah AM (2011). NADPH oxidases in cardiovascular disease: insights from in vivo models and clinical studies. Basic Res Cardiol.

[60] Staehr M, Madsen K, Vanhoutte PM, Hansen PB, Jensen BL (2011). Disruption of COX-2 and eNOS does not confer protection from cardiovascular failure in lipopolysaccharide-treated conscious mice and isolated vascular rings. Am J Physiol Regul Integr Comp Physiol.

[61] Stein B, Eschenhagen T, Rüdiger J, Scholz H, Förstermann U, Gath I (1998). Increased expression of constitutive nitric oxide synthase III, but not inducible nitric oxide synthase II, in human heart failure. J Am Coll Cardiol.

[62] Strunk V, Hahnenkamp K, Schneuing M, Fischer LG, Rich GF (2001). Selective iNOS inhibition prevents hypotension in septic rats while preserving endothelium-dependent vasodilation. Anesth Analg.

[63] Szelid Z, Pokreisz P, Liu X, Vermeersch P, Marsboom G, Gillijns H, Pellens M, Verbeken E, van de Werf F, Collen D, Janssens SP (2010). Cardioselective nitric oxide synthase 3 gene transfer protects against myocardial reperfusion injury. Basic Res Cardiol.

[64] Tavener SA, Kubes P (2006). Cellular and molecular mechanisms underlying LPS-associated myocyte impairment. Am J Physiol Heart Circ Physiol.

[65] Teng RJ, Wu TJ, Bisig CG, Eis A, Pritchard KA, Konduri GG (2011). Nitrotyrosine impairs angiogenesis and uncouples eNOS activity of pulmonary artery endothelial cells isolated from developing sheep lungs. Pediatr Res.

[66] Thielmann M, Dörge H, Martin C, Belosjorow S, Schwanke U, van de Sand A, Konietzka I, Büchert A, Krüger A, Schulz R, Heusch G (2002). Myocardial dysfunction with coronary microembolization: signal transduction through a sequence of nitric oxide, tumor necrosis factor-α, and sphingosine. Circ Res.

[67] Thiemermann C, Szabó C, Mitchell JA, Vane JR (1993). Vascular hyporeactivity to vasoconstrictor agents and hemodynamic decompensation in hemorrhagic shock is mediated by nitric oxide. Proc Natl Acad Sci USA.

[68] Tiede K, Melchior-Becker A, Fischer JW (2010). Transcriptional and posttranscriptional regulators of biglycan in cardiac fibroblasts. Basic Res Cardiol.

[69] van Faassen EE (2007) Radicals for life: the various forms of nitric oxide. Part V, pp 381–406. ISBN: 9780444522368

[70] van Faassen EE, Koeners MP, Joles JA, Vanin AF (2008). Detection of basal NO production in rat tissues using iron-dithiocarbamate complexes. Nitric Oxide.

[71] Vanin AF, Bevers LM, Mikoyan VD, Poltorakov AP, Kubrina LN, Van Faassen E (2007). Reduction enhances yields of nitric oxide trapping by iron-diethyldithiocarbamate complex in biological systems. Nitric Oxide.

[72] Vo PA, Lad B, Tomlinson JAP, Francis S, Ahluwalia A (2005). Autoregulatory role of endothelium-derived nitric oxide (NO) on Lipopolysaccharide-induced vascular inducible NO synthase expression and function. J Biol Chem.

[73] Wei XQ, Charles IG, Smith A, Ure J, Feng CJ, Huang FP, Xu DM, Muller W, Moncada S, Liew FY (1995). Altered immune-responses in mice lacking inducible nitric-oxide synthase. Nature.

[74] Zhang T, Feng Q (2010). Nitric oxide and calcium signaling regulate myocardial tumor necrosis factor-alpha expression and cardiac function in sepsis. Can J Physiol Pharmacol.

[75] Zhang T, Lu X, Li J, Chidiac P, Sims SM, Feng Q (2012). Inhibition of Na/K-ATPase promotes myocardial tumor necrosis factor-alpha protein expression and cardiac dysfunction via calcium/mTOR signaling in endotoxemia. Basic Res Cardiol.

[76] Zhang YS, He L, Liu B, Li NS, Luo XJ, Hu CP, Ma QL, Zhang GG, Li YJ, Peng J (2012). A novel pathway of NADPH oxidase/vascular peroxidase 1 in mediating oxidative injury following ischemia-reperfusion. Basic Res Cardiol.

