# Supplementary material for: Endothelial NOS (NOS3) impairs myocardial function in developing sepsis
Source: Basic Res Cardiol. 2013 Feb 10;108(2):330. doi: 10.1007/s00395-013-0330-8 (PMC3597270; doi:10.1007/s00395-013-0330-8)
Supplement: Supplementary file 1 — Supplementary material 1 (PDF 13178 kb) [file 395_2013_330_MOESM1_ESM.pdf]

Online Resource 1

Article title:
Endothelial NOS (NOS 3) impairs myocardial function in developing sepsis

Journal name:
Basic Research in Cardiology

Author names:
Annette M. van de Sandt, MD; Rainer Windler, MSc; Axel Gödecke, PhD, Jan Ohlig, MD; Simone Zander MSc, Michael Reinartz, MSc, Jürgen Graf, MD; Ernst E. van Faassen, PhD; Tienush Rassaf, MD; Jürgen Schrader, MD; Malte Kelm, MD; Marc W. Merx, MD

Corresponding author:
Marc W. Merx. M.D.
Department of Medicine
Division of Cardiology, Pneumology and Angiology
Moorenstrasse 5, D- 40225 Düsseldorf
Phone: +49 (0) 211- 8118801, Fax: +49 (0) 211- 8118812
Email: marc.merx@med.uni-duesseldorf.de

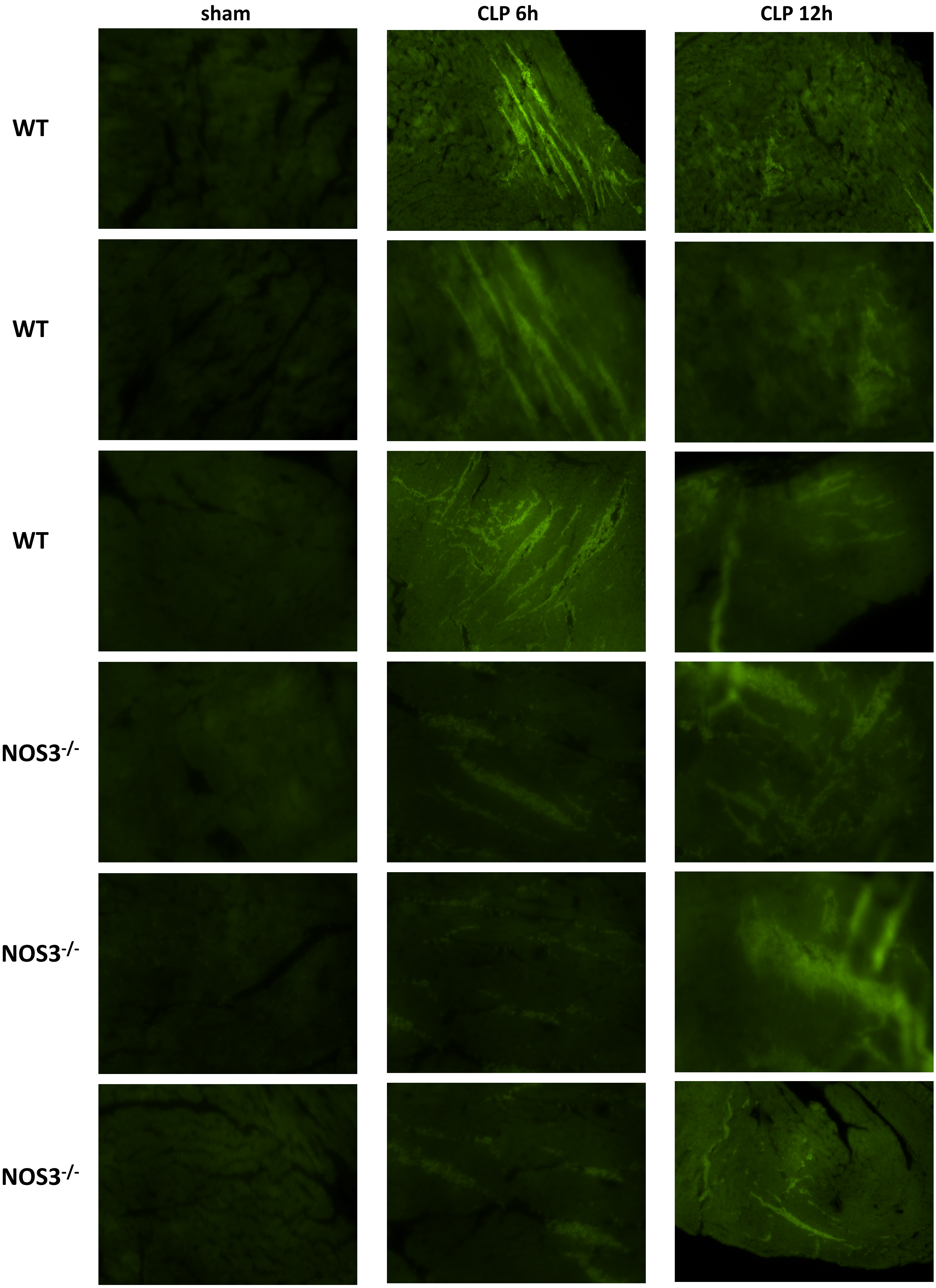

Online Resource 1

Figure legend.

Online Resource 1. NOS3 increases 3-nitrotyrosine content in heart tissue in developing sepsis. Immunohistochemical staining demonstrated an increase of nitrotyrosine in heart tissue of septic wildtype mice at 6 hours post CLP, whereas no differences could be detected in $NOS3^{-/-}$ mice.

Methods Online Resource 1.

(Methods description included in main manuscript, reproduced here for convenience)

Immunohistochemistry. Heart samples were fixed in 4% formaldehyde phosphate buffer, dehydrated and paraffin-embedded. For 3-nitrotyrosin detection serial sections (3 per mouse, 400µm apart) were incubated with a polyclonal antibody raised against nitrotyrosine (USBiological, N2700-06), followed by sheep anti-rabbit IgG conjugated to FITC (Rockland, 611-641-122).
