# Supplementary material for: Endothelial NOS (NOS3) impairs myocardial function in developing sepsis
Source: Basic Res Cardiol. 2013 Feb 10;108(2):330. doi: 10.1007/s00395-013-0330-8 (PMC3597270; doi:10.1007/s00395-013-0330-8)
Supplement: Supplementary file 2 — Supplementary material 2 (PDF 1273 kb) [file 395_2013_330_MOESM2_ESM.pdf]

Online Resource 2

Article title:
Endothelial NOS (NOS 3) impairs myocardial function in developing sepsis

Journal name:
Basic Research in Cardiology

Author names:
Annette M. van de Sandt, MD; Rainer Windler, MSc; Axel Gödecke, PhD, Jan Ohlig, MD; Simone Zander MSc, Michael Reinartz, MSc, Jürgen Graf, MD; Ernst E. van Faassen, PhD; Tienush Rassaf, MD; Jürgen Schrader, MD; Malte Kelm, MD; Marc W. Merx, MD

Corresponding author:
Marc W. Merx. M.D.
Department of Medicine
Division of Cardiology, Pneumology and Angiology
Moorenstrasse 5, D- 40225 Düsseldorf
Phone: +49 (0) 211- 8118801, Fax: +49 (0) 211- 8118812
Email: marc.merx@med.uni-duesseldorf.de

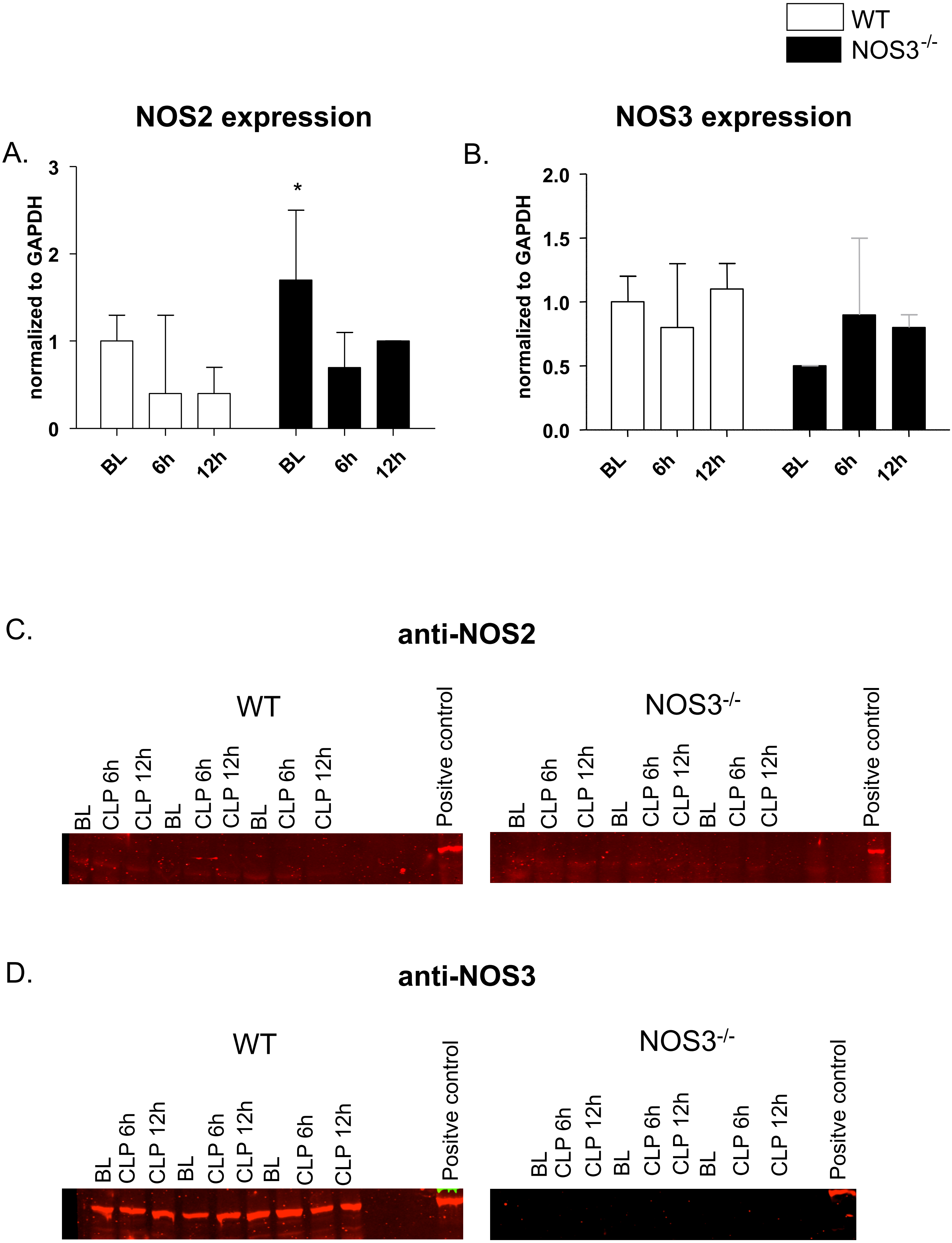
WT
NOS3-/-
NOS2 expression
A.
normalized to GAPDH
3
2
1
0
*
BL
6h
12h
BL
6h
12h
NOS3 expression
B.
normalized to GAPDH
2.0
1.5
1.0
0.5
0.0
BL
6h
12h
BL
6h
12h
C.
anti-NOS2
WT
BL
CLP 6h
CLP 12h
BL
CLP 6h
CLP 12h
BL
CLP 6h
CLP 12h
Positve control
NOS3-/-
BL
CLP 6h
CLP 12h
BL
CLP 6h
CLP 12h
BL
CLP 6h
CLP 12h
Positve control
D.
anti-NOS3
WT
BL
CLP 6h
CLP 12h
BL
CLP 6h
CLP 12h
BL
CLP 6h
CLP 12h
Positve control
NOS3-/-
BL
CLP 6h
CLP 12h
BL
CLP 6h
CLP 12h
BL
CLP 6h
CLP 12h
Positve control

E.

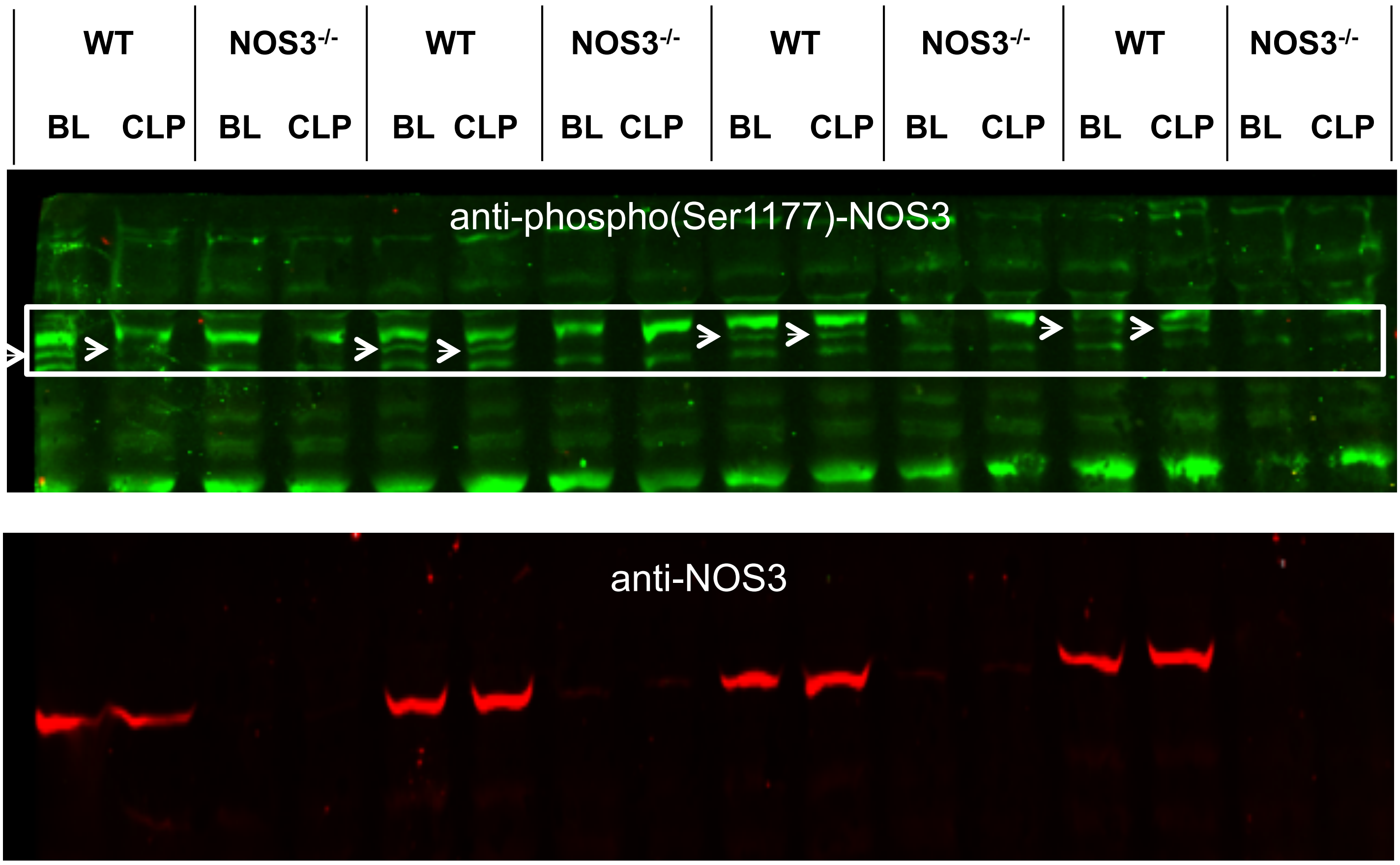

Online Resource 2.

Figure legend.

Online Resource 2. 6 and 12 hours after sepsis induction revealed neither increase in NOS2- nor NOS3-expression in septic WT and NOS3$^{-/-}$ mice compared to baseline (A+B). Cardiac protein expression of NOS2 remained below detection levels in both strains (C). NOS3 protein expression did not differ in septic WT mice at 6 and 12 hours post CLP compared to baseline. Absence of NOS3 (eNOS) protein in NOS3$^{-/-}$ mice (D). No signficant increase in phospho-NOS3 in septic WT mice compared to baseline (E).

Methods Online Resource 2.

RNA extraction and Quantitative RT-PCR analysis. Total RNA was extracted from mouse hearts by using “RNeasy® fibrous tissue kit” (Qiagen). Total RNA (1µg per sample) was reverse-transcribed, and real-time PCR was performed in triplicate using the Applied Biosystems 7300 Fast Real-time PCR system and TaqMan® GenExpression Assays (Applied Biosystems) for NOS3 (nitric oxide synthase 3, endothelial; Mm00435217_m1), NOS2 (nitric oxide synthase 2, inducible; Mm00440502_m1), NOS1 (nitric oxide synthase 1, neuronal; Mm00435175_m1), and GAPDH as endogenous control (glyceraldehyde-3-phosphate dehydrogenase; Mm99999915_g1). The setup of reaction consisted of 1 µl of cDNA (10ng), 1 µl of TaqMan primer set, 10 µl Taq [TaqMan® Gen Expression Master Mix (2x); no. 4369016], and 8 µl of H_2O. PCR was performed according to manufacture´s instructions (standard run type).

Measurements of phosho-NOS3, NOS3, NOS2, and NOS1 protein. Protein extracts were prepared by homogenization of mouse tissues in 50 mmol/L Tris (pH 7.5), 1 mmol/L EDTA, and 1 mmol/L PMSF. Extracts were centrifuged for 10 minutes at 10000 rpm. Supernatant protein was separated on 7,5% SDS polyacrylamide gels and electroblotted to nitrocellulose. Western Blot analyses were performed as previously described (1). Primary antibodies: phosho-eNOS/NOS3 (Ser 1177) (Cell Signaling 9571S), eNOS/NOS3 (Transduction 610296), iNOS/NOS2 (Transduction N32020), nNOS/NOS1 (Transduction N31020). Secondary antibodies: IRDye goat anti-mouse or goat anti-rabbit (Licor). Antibodies were used according to manufacturer´s instructions and signals were detected with the LI-COR Odyssey Infrared Imaging System and the Odyssey Software V3.0.

Reference:

1. Gödecke A; Circ Res. 1998;82:196
