# Supplementary material for: Endothelial NOS (NOS3) impairs myocardial function in developing sepsis
Source: Basic Res Cardiol. 2013 Feb 10;108(2):330. doi: 10.1007/s00395-013-0330-8 (PMC3597270; doi:10.1007/s00395-013-0330-8)
Supplement: Supplementary file 3 — Supplementary material 3 (PDF 795 kb) [file 395_2013_330_MOESM3_ESM.pdf]

Online Resource 3

Article title:
Endothelial NOS (NOS 3) impairs myocardial function in developing sepsis

Journal name:
Basic Research in Cardiology

Author names:
Annette M. van de Sandt, MD; Rainer Windler, MSc; Axel Gödecke, PhD, Jan Ohlig, MD; Simone Zander MSc, Michael Reinartz, MSc, Jürgen Graf, MD; Ernst E. van Faassen, PhD; Tienush Rassaf, MD; Jürgen Schrader, MD; Malte Kelm, MD; Marc W. Merx, MD

Corresponding author:
Marc W. Merx. M.D.
Department of Medicine
Division of Cardiology, Pneumology and Angiology
Moorenstrasse 5, D- 40225 Düsseldorf
Phone: +49 (0) 211- 8118801, Fax: +49 (0) 211- 8118812
Email: marc.merx@med.uni-duesseldorf.de

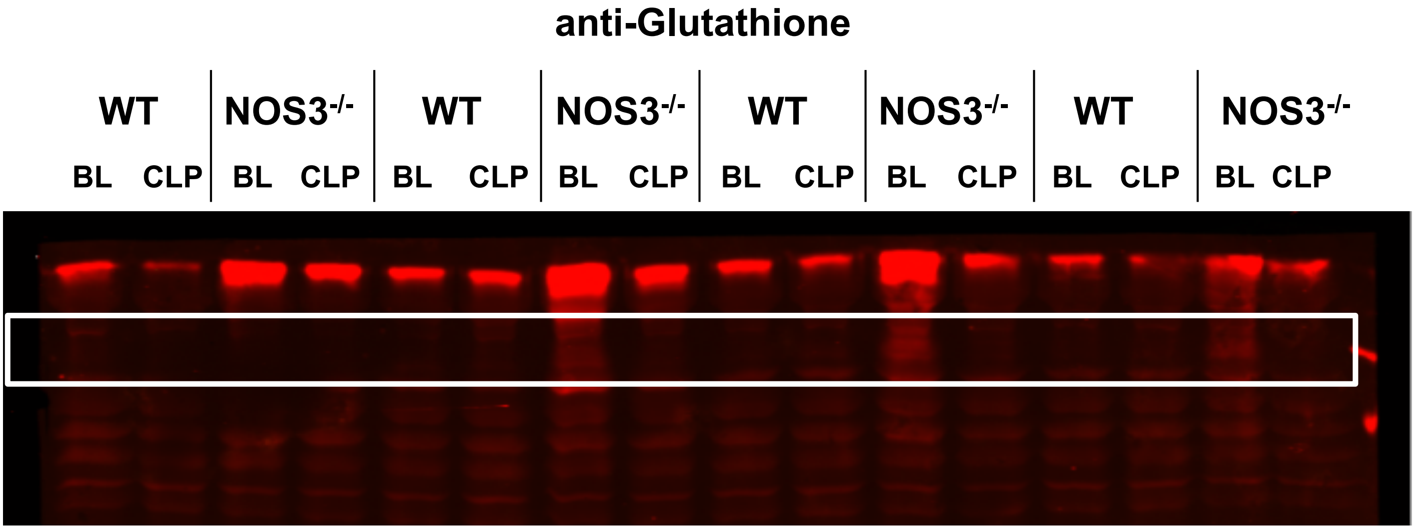

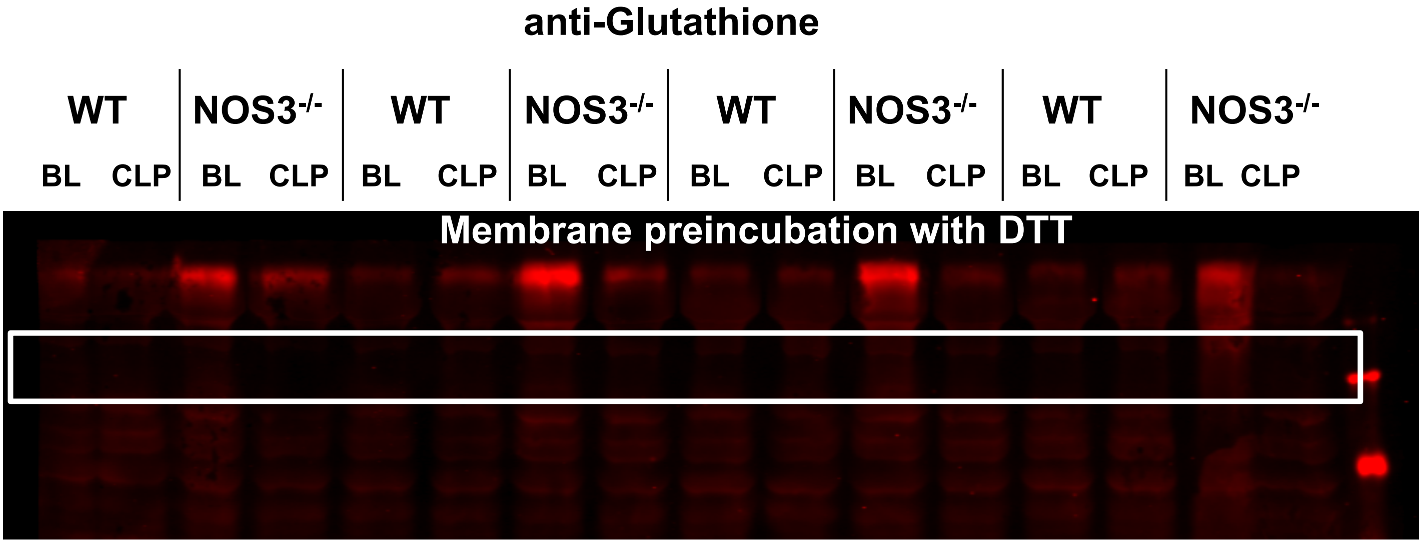

Online Resource 3

Figure legend.

Online Resource 3. Global protein glutathionylation. Protein glutathionylation was not increased in sepsis developing mice. In WT mice no increase in glutathionylation could be observed at the level of eNOS/ NOS3.

Methods Online Resource 3.

eNOS/NOS3 glutathionylation. After in situ perfusion with NaCl supplemented with N-ethylmaleimide (NEM)/EDTA hearts were excised from the mice, snap frozen in liquid nitrogen and homogenized in N-ethylmaleimide (40 mM) and SDS (2%) containing HEPES (50 mM, pH7.0) buffer. NEM irreversibly alkylates thiol groups to prevent further S-glutathionylation during sample processing. Protein extracts were separated by SDS-PAGE under non reducing conditions and blotted onto nitrocellulose membranes. S-glutathionylated proteins were detected by Western Blot using an anti-glutathione monoclonal antibody (Thermo Scientific MA1-7620). As negative control the Western Blot membrane was pre-incubated with dithiothreitol (DTT).
