# Supplementary material for: Endothelial NOS (NOS3) impairs myocardial function in developing sepsis
Source: Basic Res Cardiol. 2013 Feb 10;108(2):330. doi: 10.1007/s00395-013-0330-8 (PMC3597270; doi:10.1007/s00395-013-0330-8)
Supplement: Supplementary file 4 — Supplementary material 4 (PDF 71 kb) [file 395_2013_330_MOESM4_ESM.pdf]

Online Resource 4.

Article title:
Endothelial NOS (NOS 3) impairs myocardial function in developing sepsis

Journal name:
Basic Research in Cardiology

Author names:
Annette M. van de Sandt, MD; Rainer Windler, MSc; Axel Gödecke, PhD, Jan Ohlig, MD; Simone Zander MSc, Michael Reinartz, MSc, Jürgen Graf, MD; Ernst E. van Faassen, PhD; Tienush Rassaf, MD; Jürgen Schrader, MD; Malte Kelm, MD; Marc W. Merx, MD

Corresponding author:
Marc W. Merx. M.D.
Department of Medicine
Division of Cardiology, Pneumology and Angiology
Moorenstrasse 5, D- 40225 Düsseldorf
Phone: +49 (0) 211- 8118801, Fax: +49 (0) 211- 8118812
Email: marc.merx@med.uni-duesseldorf.de

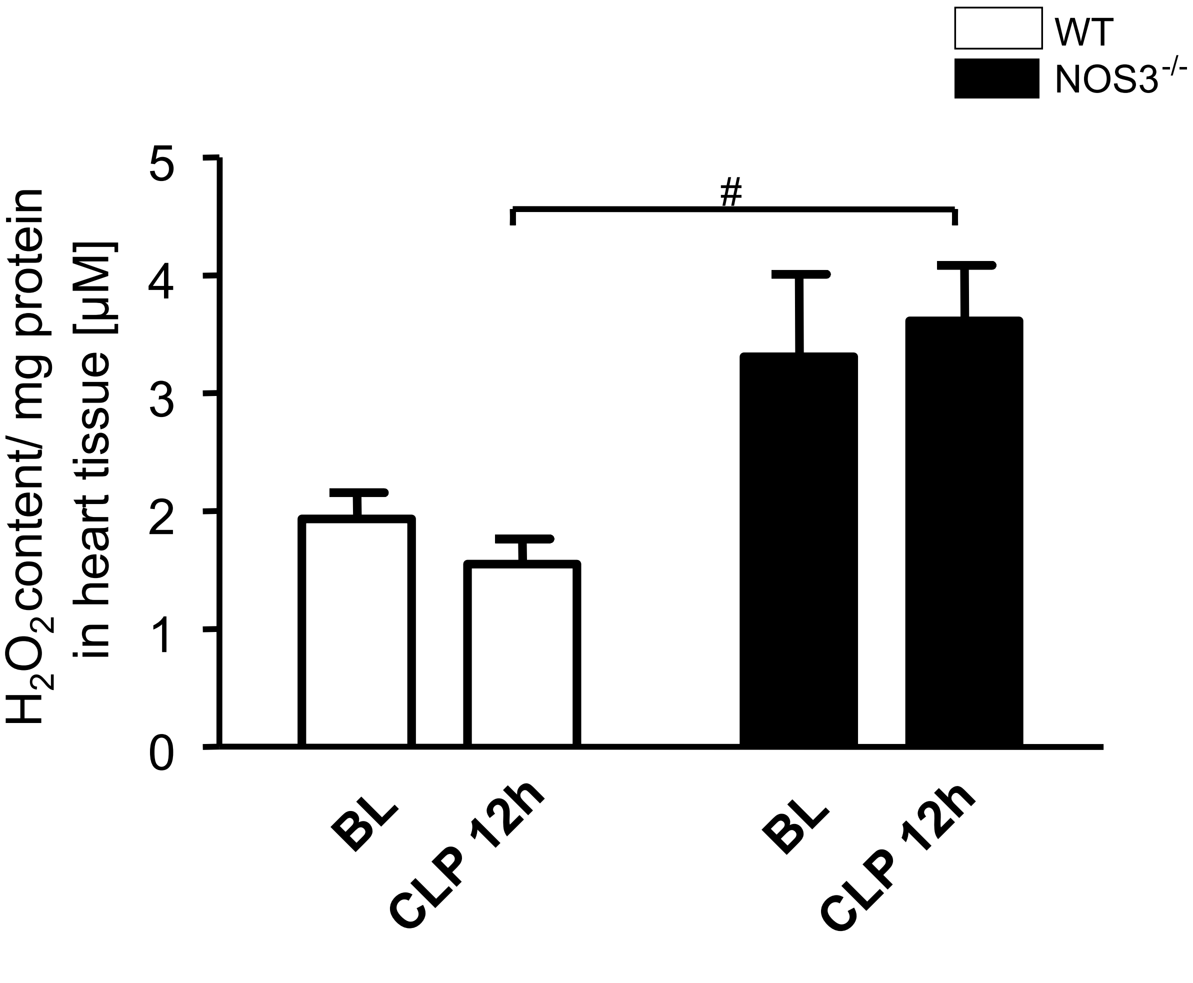

Online Resource 4

Figure legend.

Online Resource 4. No signs for significant oxidative stress in heart tissue in developing sepsis. Fluorometrically determined H_2O_2 generation in cardiac tissues demonstrated no increase in H_2O_2 generation in developing sepsis compared to baseline in both strains. $NOS3^{-/-}$ mice exhibited more ROS generation at baseline and post CLP compared to WT mice most probably because of basal NO-deficiency (#P<0.05 WT vs. $NOS3^{-/-}$ mice; n=4-5 per group).

Methods Online Resource 4.

H_2O_2 Assay. ROS generation (H_2O_2) in cardiac tissue was determined fluorometrically by monitoring the oxidation of amplex red reagent (10-acetyl-3,7-dihydroxyphenoxazine) to the product resorufin using the Amplex Red Hydrogen Peroxide/ Peroxidase assay kit (Invitrogen, Karlsruhe, Germany) according to manufactor´s instruction. Fluorescence was measured with a fluorescence microplate reader (Floustar Omega, BMG Labtech, Ortenberg, Germany) using excitation at 544 nm and fluorescence emission detection at 590 nm. Background fluorescence, determined for a no H_2O_2-control reaction has been subtracted from each value.
