# Supplementary material for: Endothelial NOS (NOS3) impairs myocardial function in developing sepsis
Source: Basic Res Cardiol. 2013 Feb 10;108(2):330. doi: 10.1007/s00395-013-0330-8 (PMC3597270; doi:10.1007/s00395-013-0330-8)
Supplement: Supplementary file 5 — Supplementary material 5 (PDF 113 kb) [file 395_2013_330_MOESM5_ESM.pdf]

Online Resource 5. Isometric force measurements in aortic rings of septic wildtype compared to septic NOS3$^{-/-}$ mice

Article title:
Endothelial NOS (NOS 3) impairs myocardial function in developing sepsis

Journal name:
Basic Research in Cardiology

Author names:
Annette M. van de Sandt, MD; Rainer Windler, MSc; Axel Gödecke, PhD, Jan Ohlig, MD; Simone Zander MSc, Michael Reinartz, MSc, Jürgen Graf, MD; Ernst E. van Faassen, PhD; Tienush Rassaf, MD; Jürgen Schrader, MD; Malte Kelm, MD; Marc W. Merx, MD

Corresponding author:
Marc W. Merx. M.D.
Department of Medicine
Division of Cardiology, Pneumology and Angiology
Moorenstrasse 5, D- 40225 Düsseldorf
Phone: +49 (0) 211- 8118801, Fax: +49 (0) 211- 8118812
Email: marc.merx@med.uni-duesseldorf.de

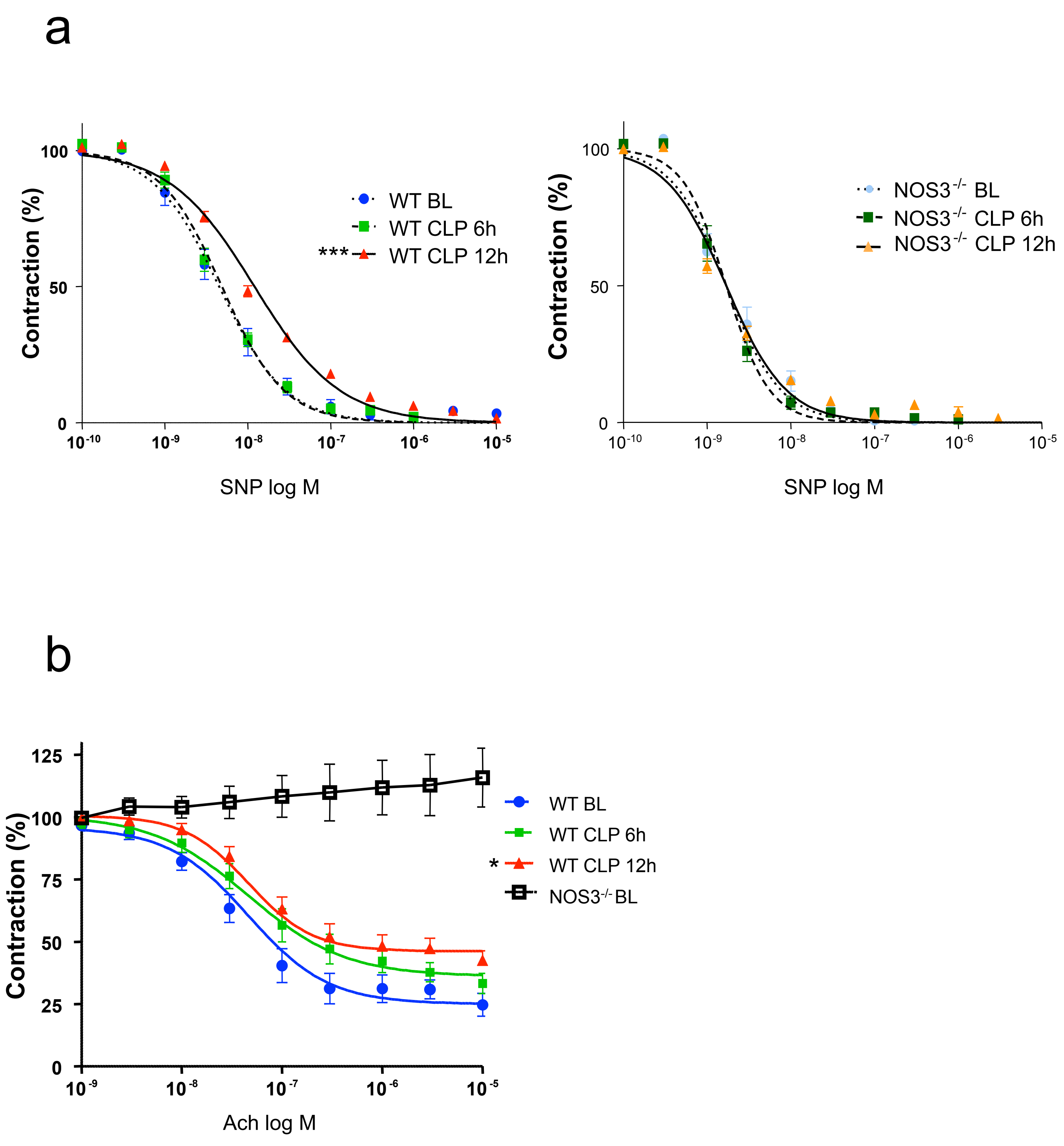

Online Resource 5

Figure legend.

Online Resource 5. NOS3 reduces sensitivity to NO donor in ongoing sepsis as a result of high bioactive NO (A). Segments of thoracic aorta from wildtype and $NOS3^{-/-}$ mice were isolated at baseline, 6 and 12 hours post CLP and subsequently suspended in a myography. After application of SNP isometric force measurements in isolated aortic rings demonstrated a significantly reduced sensitivity to SNP in septic wildtype mice at 12 hours post CLP. Sensitivity to SNP remained stable in $NOS3^{-/-}$ during all phases (n= 6-15 per group, ***P< 0.001 vs baseline wildtype mice). **NOS3 impairs endothelial function in developing sepsis (B).** While $NOS3^{-/-}$ mice demonstrated no response to acetylcholine, as it would be expected, septic wildtype mice exhibited significant endothelial dysfunction at 12 hours of sepsis (*P< 0.05 vs baseline wildtype mice)

Methods Online Resource 5.

Isometric force measurements in aortic rings. Thoracic aorta was removed as previously described at baseline, 6 and 12 hours post CLP [1]. Aortic rings were mounted on a myograph, under 1 g of tension, and bathed in 2 mL of Kreb’s buffer constantly gassed with 95% O_2/5% CO_2 at 37 °C. After equilibration phase (90 minutes) tissues were exposed to potassium chloride (80 m) and subsequently phenylephrine (1µM) to achieve maximal contraction. Relaxation response curves to acetylcholine were determined as the response of EC50 to acetylcholine. After precontraction with phenylephrine again, relaxation response curves to increasing concentrations (0,001-10µM) of the NO donor sodium nitroprusside (SNP) were constructed.

Reference

1. Owen PJ, Ying H, Lang D, Tomlinson D, Lewis MJ, Cheng SY, Lazarus JH (2007) Endothelial dysfunction in a murine model of thyroid hormone resistance. Eur J Clin Invest 37:390-395 doi:10.1111/j.1365-2362.2007.01799.x.
